# Optical Coherence Tomography Parameters Related to Vision Impairment in Patients with Diabetic Macular Edema: A Quantitative Correlation Analysis

**DOI:** 10.1155/2020/5639284

**Published:** 2020-09-27

**Authors:** Bing Li, Bilei Zhang, Youxin Chen, Donghui Li

**Affiliations:** Department of Ophthalmology, Peking Union Medical College Hospital, Key Laboratory of Ocular Fundus Diseases, Chinese Academy of Medical Sciences, Beijing, China

## Abstract

**Purpose:**

To quantitatively explore the correlation between optical coherence tomography (OCT) parameters and vision impairment in patients with diabetic macular edema (DME).

**Methods:**

This study was a retrospective observational case series. One-hundred eyes from 66 patients with DME were retrospectively included. OCT parameters, including central macular thickness (CMT), height of intraretinal cystoid, subretinal fluid and sponge-like retinal swelling, density of hyperreflective foci (HRF), and integrity of the ellipsoidal zone (EZ), were assessed. Correlation analyses and multiple linear regression analysis were performed to quantitatively explore the relationship between best-corrected visual acuity (BCVA) and OCT parameters.

**Results:**

Among all OCT parameters, CMT, height of intraretinal cystoid, height of sponge-like retinal swelling, and density of HRF and EZ integrity were significantly correlated with BCVA (*r* = −0.550, −0.526, −0.411, −0.277, and −0.501, respectively; *P* < 0.01). In multiple linear regression analysis, CMT, density of HRF, and EZ integrity fit a significant linear equation (*β* = 0.482, 0.184, and 0.447, respectively), with the adjusted *R* square reaching 0.522 (*P* < 0.001). In eyes without SRF, the height of intraretinal cystoid, density of HRF, and EZ integrity were included in the model and an adjusted *R* square of 0.605 (*P* < 0.001) was obtained.

**Conclusion:**

In DME eyes, OCT parameters, including the density of HRF, the EZ integrity together with CMT, or the height of intraretinal cystoid, could explain 52.2% to 60.5% of the variation in BCVA and were weighted approximately 2 : 1 : 2, respectively.

## 1. Introduction

Diabetic macular edema (DME) is the most common cause of vision impairment in patients with diabetic retinopathy (DR) and affects approximately 7% of all patients with diabetes [1, 2]. Noninvasive optical coherence tomography (OCT) has been used to assess DME and to evaluate the efficacy of treatment as it provides reliable high-resolution imaging of retinal anatomy and quantification of central macular thickness (CMT) [3]. However, previous investigations demonstrated that CMT presented only a modest correlation with visual acuity (VA) and might not be an appropriate surrogate marker of VA [4, 5], which is influenced by various factors other than CMT [6]. For example, hyperreflective foci (HRF) have been considered a precursor of hard exudate (HE) and could be a possible new surrogate marker related to foveal photoreceptor damage [7]. The disturbance of the ellipsoid zone (EZ) represents damage to photoreceptors and concomitant visual disturbance [8]. Also, previous studies demonstrated that DR patients have higher levels of circulating inflammatory cytokines and vascular endothelial growth factor (VEGF) compared with patients without retinopathy which also presented a correlation with OCT signs such as the integrity of EZ [9, 10].

In fact, OCT presents various morphologic characteristics in DME, such as subretinal fluid (SRF) and intraretinal cystoid, in addition to CMT. The pathophysiology of these morphologic characteristics is quite different [11–13]. Previous studies classified DME into different types, diffuse retinal thickening (DRT), cystoid macular edema (CME), and serous retinal detachment (SRD), according to their morphological characteristics on OCT [5]. However, DME often presents as combinations of multiple types such that clear classification is difficult in some cases. In addition to retinal thickness parameters, other OCT signs, such as the presence of HRF and the discontinuous EZ, also indicate the severity of the disease and retina damage. Some studies have investigated the correlation between VEGF level and OCT signs as well as different OCT parameters and VA outcomes and explored their predictive values in treatment [10]. However, assessments of the impact these parameters have on visual impairment remain very rare.

In this study, we investigated the correlation between different OCT parameters and VA to estimate their impact on vision impairment and explored their correlation through quantitative analysis.

## 2. Methods

This was a retrospective case series study. All the research methods and measurements adhered to the tenets of the Declaration of Helsinki and had the approval of the Ethics Committee of Peking Union Medical College Hospital for retrospective review of existing patients' data.

### 2.1. Study Participants

We retrospectively reviewed the medical records of 100 eyes (66 patients) with DME from January 1, 2013, to August 1, 2015, at Peking Union Medical College Hospital.

All patients had undergone comprehensive ophthalmologic examinations, including best-corrected visual acuity (BCVA) measured on the Early Treatment Diabetic Retinopathy Study (ETDRS) chart, slit-lamp biomicroscopy, color fundus photography (CFP), spectral domain (SD) OCT, and fluorescence fundus angiography (FFA) at their first visit. In addition, the duration of diabetes mellitus (DM) and the most recent glycosylated hemoglobin (GHb) level, previous panretinal photocoagulation (PRP), and other ophthalmic history or treatments were recorded.

To be included in the analysis, patients had to fulfill the following inclusion criteria: (1) age ≥ 18 years, (2) type 1 or type 2 DM, (3) visual loss caused by center-involved DME (OCT measured CMT ≥ 300 *μ*m), and (4) nonproliferative diabetic retinopathy (NPDR). The exclusion criteria were as follows: (1) significant media opacity that precluded adequate images, (2) cataract surgery within 6 months, (3) intravitreal anti-VEGF agents (bevacizumab, ranibizumab, aflibercept, or conbercept) within 3 months or intravitreal steroid therapy (triamcinolone or dexamethasone) within 6 months, (4) previous vitrectomy, and (5) other nondiabetic pathologies that might substantially affect VA.

### 2.2. Optical Coherence Tomography Analysis

The OCT images of the macula were obtained using SD OCT (Spectralis HRA + OCT, Heidelberg Engineering, Heidelberg, Germany) with a standard imaging protocol consisting of 49 B-scans spanning a 20 × 20 frame, with a mean of 9 automatic real-time images per scan in high-resolution mode and a radioactive scanning mode with 6 B-scans centralized by the fovea. The morphologic features were evaluated within a radius of 1000 *μ*m centralized by the fovea.

The OCT parameters were obtained for qualitative assessment, including (1) CMT calculated automatically by the instrument; (2) height of the SRF (measured right beneath the fovea); (3) height of intraretinal cystoid at the outer nuclear layer (ONL); the maximum height of the cystoid perpendicular to the retinal pigmented epithelium (RPE) layer was measured; (4) sponge-like retinal swelling; the maximum range from inner plexiform layer to the RPE layer was measured; (5) density of HRF (few 2–10, moderate 11–20, many > 21–20, dense > 21) [14]; and (6) the EZ integrity was defined and recorded as regular and continuous, partial irregularity, and lack of continuity and complete discontinuity [15]. The OCT parameters were measured by two independent observers (Li B and Zhang BL). An experienced retina specialist (Chen YX) was invited for the estimation of contradictions. The interobserver correlation was assessed using Pearson correlation coefficients for the agreement of OCT paramotors ranging from 0.78 to 0.84.

### 2.3. Statistical Analysis

SPSS 23.0 (IBM SPSS Statistics for Windows, Version 23.0. Armonk, NY: IBM Corp) was used as the statistical analysis software for the assessments performed in this study. The Kolmogorov–Smirnov test was performed to test the normality of the continuous variable. Pearson correlation analysis was performed on normal continuous variables, and Spearman correlation analysis was used for categorical variables. With BCVA as the dependent variable, variables that reached significance in the preceding steps were then tested using multiple linear regression analysis to quantitatively explore the correlation between BCVA and the selected OCT parameters. The backward entered method was adopted, and parameters were excluded if the *P* value exceeded 0.10. Since the height of SRF accounts for a large proportion of CMT, a subgroup analysis of DME with or without SRF was performed. We considered a *P* value of 0.05 or less to be statistically significant in this study.

## 3. Results

A total of 100 eyes (66 patients) were included in the study, with the mean age of 57.3 ± 10.5 years. Descriptive characteristics of the enrolled patients are shown in Table 1. No significant differences were detected between BCVA in DME eyes with or without PRP and between phakic or pseudophakic eyes (*P* values were 0.371 and 0.569).

### 3.1. Correlation Assessment between OCT Parameters and BCVA

CMT, the height of the intraretinal cystoid, the height of the sponge-like retinal swelling, and the EZ integrity showed a moderate negative correlation with BCVA (*r* = −0.550, −0.526, and −0.501, respectively, *P* < 0.001), while the density of HRF showed a mild negative correlation (*r* = −0.277, *P* value was 0.005). SRF did not present a significant correlation with BCVA (*P* value was 0.095).

Subgroup analysis in eyes without SRF, the height of the intraretinal cystoid, and the EZ integrity presented a stronger correlation with BCVA in the subgroup analysis compared with that in the ungrouped analysis (*r* = −0.698 and −0.685, respectively, *P* < 0.001). Other parameters, including CMT, the height of sponge-like retinal edema, and the density of HRF, also showed significant negative correlations (*r* = −0.617, −0.544, and −0.270, respectively; *P* < 0.05). In eyes with SRF, only CMT presented a moderate correlation with BCVA (*r* = −0.472, *P* value was 0.003). Other parameters were not significant (Table 2).

### 3.2. Quantitative Analysis of OCT Parameters and BCVA

We included five OCT parameters, which reached significance in the previous analysis, into the multivariate regression linear analysis. They were the CMT, height of intraretinal cystoid and sponge-like retinal swelling, density of HRF, and EZ integrity. The CMT, density of HRF, and the EZ integrity were included and obtained an equation with an adjusted *R* square reaching 0.522 (*P* < 0.001), which indicated that the regression equation could explain 52.2% of the variation in BCVA.

Subgroup analysis in eyes without SRF and the height of intraretinal cystoid were included instead of CMT, the density of HRF and the EZ integrity were also included, and the adjusted *R* square reached 0.605 (*P* < 0.05), indicating that the model could explain 60.5% of BCVA variation. In DME eyes with SRF, only CMT was included in the linear regression model and obtained an adjusted *R* square of 0.201 (*P*=0.003), meaning that the regression equations were significant but could explain only 20.1% of the variation in BCVA.  Table 3.

## 4. Discussion

In the present study, the OCT parameters we selected represent different pathomechanisms. The swollen and thickened central retina results from vascular leakage and broke down blood-retinal barrier [3]. The pathogenesis of SRF remains unclear, but current research holds the opinion that the fluid pressure from the vascular leakage plays an important role [16]. HRF correlates with the development of intraretinal HEs and implies active inflammation [17]. The EZ reportedly represents the status of photoreceptors [8]. In the multiple linear regression analysis, with the parameters of CMT, density of HRF, and EZ integrity, the model could explain 52.2% of the variation in BCVA. However, in eyes without SRF, the height of the intraretinal cystoid was considered a better parameter to fit the model instead of CMT, and the model could explain over 60% of BCVA variation. Since the three included parameters represent different pathogeneses, we consider the model to be statistically significant as well as reasonable from an ophthalmic perspective. According to the results of the multiple linear regression models, the weight of different parts (CMT/height of the intraretinal cystoid, density of HRF, and EZ integrity) is approximately 2 : 1 : 2, which indicates that the degree of retinal thickness (CMT or the height of intraretinal cystoid) could explain only a small part of visual impairment.

CMT has been considered an extremely important index in most studies of DME. In our study, CMT showed a moderate correlation with BCVA, which is consistent with that of the previous studies [5]. Considering that CMT may not represent the swollen status of the local retina or different layers precisely, we further explored the correlation between BCVA and different types of retinal thickness. The height of intraretinal cystoids and sponge-like retinal swelling were both moderately correlated with BCVA. SRF did not seem to be correlated with BCVA, which was also proven in the previous studies [18]. Although the pathogenesis of SRF remains unclear, compared with eyes without SRF, DME eyes are considered to have greater severity and more active inflammation of the disease with higher VEGF and interleukin-6 levels [19, 20]. Intraretinal cystoids are considered to be located primarily in the outer retinal layers in the former stage and involve almost the entire retinal layer, leaving a thin outer layer in the fovea in the later stage [5]. Therefore, the large intraretinal cystoid implies a longer duration and greater disorganized retinal structure with the progression of the disease, which will result in poor visual outcomes. Murakami et al. [21] reported a correlation of intraretinal cystoid space with the interruption of the EZ beneath indicates that it could also influence visual function by the disorganization of the outer retina. However, in eyes with SRF, the height of intraretinal cysts was not significantly correlated with BCVA. This is also reasonable since SRF may account for a large part of CMT, sometimes even greater than the thickness of the whole sensory retina. The accumulated fluid also pushes the retina inward and makes it thinner than normal because of tension from stretching. In addition, more disorganized morphologic features on OCT scans seem to have a more complex influence on BCVA. Subgroup analysis showed that only CMT was significantly correlated with BCVA in eyes with SRF. In the present paper, the sponge-like retinal swelling was considered a uniform thickening of the outer layer or the whole retina. Although this swelling also presented a significant correlation with BCVA, it was excluded because of a nonsignificant contribution to the model regardless of the whole group or subgroup analysis. One reason may be that CMT and the height of intraretinal cystoid could represent its effects to some extent. Additionally, most of the sponge-like retinal lesions were not very close to the fovea (less than 500 *μ*m to the fovea).

HRF is a morphological sign of accumulation of lipid extravasation, proteinaceous material, and inflammatory cells and consequently precursors of HEs [22]. The correlation of HRF with VA is controversial in different studies. Uji et al. [7] demonstrated that the presence of HRF in the outer retina was associated with a decreased VA [7]. However, another study proved that baseline HE was not meaningfully correlated with baseline or changes in VA after treatment with ranibizumab [23]. The different methods adopted in these studies may partially explain their different results. In our study, we used SD OCT to study the density of HRF within 1000 *μ*m around the fovea to minimize the variation. However, its relatively random distribution makes quantitative analysis difficult. Instead, a severity score classification was adopted to analyze its effect and found a mild correlation with BCVA. According to previous studies, HRF is associated with a disrupted external limiting membrane or EZ, suggesting photoreceptor degeneration in patients with DME [7]. This parameter also represents the accumulation of inflammatory cells in DR [24, 25]. However, the HRF of the B-scan was dense and difficult to accurately quantify. We analyzed the density of HRF through a severity score using the study of Zur et al. [14] for reference and found a mild correlation with VA. Further studies with a more accurate quantification system, especially artificial intelligence, might help find a precise association between HRF and BCVA.

The disturbance of the EZ has been considered to reflect anatomical disruptions of the photoreceptors. The EZ might represent cellular damage or cell death [26, 27], either of which would explain the wide range of VA for a given degree of retinal edema. Previous studies also found a correlation between the integrity of EZ and visual function [28–31]. Shen et al. reported that the EZ integrity is more strongly correlated with BCVA than with CMT [29]. The present study showed the comparative weight of CMT and the EZ integrity in the linear regression analysis. The severity of EZ disturbance increased by one grade, approximately equivalent to a 100 *μ*m increase in CMT. We attribute the differences to the severity of DR of the enrolled patients. According to the previous studies, the ischemic condition of both the retina and choroid begins at the very early stage of DR, which may affect the function of photoreceptors immediately [32]. The DME eyes enrolled in this study were all at the stage of NPDR. With the progression of the disease and macular ischemia, the damage to photoreceptors becomes even greater and the EZ integrity may be even more strongly correlated with visual outcomes. Also, other factors presented effects on the integrity of EZ. As previous reported, an increase in serum VEGF, intercellular cell adhesion molecule (ICAM)-1, and antimyeloperoxidase antibody levels are associated with an increase in the severity of diabetic retinopathy and the grade of external limiting membrane and EZ disruption as well as decreased visual acuity in diabetic retinopathy.

In this study, we analyzed the importance of the quantitative correlation with OCT parameters and BCVA. We found a significant regression model to explore their impact on visual impairment. To our knowledge, this is the first study to state the quantitative correlation and OCT parameters with BCVA and their impact on visual impairment in DME patients with NPDR. The limits of this study were its retrospective design and small sample size. We used a classification system to assess the integrity of EZ and the severity of HRF for reference in consideration of clinical use, but it may also not be precise for further analysis. Other assessment techniques, such as artificial intelligence, are needed for precise analysis of these two parameters.

## Figures and Tables

**Table 1:** Descriptive characteristics of the enrolled patients and the OCT parameters.

Characteristics	Value
Age (yrs), mean ± SD	57.3 ± 10.5
Gender, male (%)	37 (56.1)
Duration of diabetes mellitus (yrs), mean ± SD	12.2 ± 7.0
Previous PRP, *N* (%)	
PRP	53 (53)
Non-PRP	47 (47)
Lens status	
Phakic, *n* (%)	82 (82)
Pseudophakic	18 (18)
BCVA (ETDRS letters), mean ± SD	58.2 ± 12.2
OCT parameters	
Central retinal thickness	522.5 ± 168.5
Subretinal fluid	
*N* (%)	38 (38%)
Height, mean ± SD	191.5 ± 131.7
Intraretinal cysts	
*N* (%)	97 (97%)
Height, mean ± SD	343.8 ± 167.6
Sponge-like retinal swelling	
*N* (%)	80 (80%)
Height, mean ± SD	377.5 ± 100.2
HRF, *N*	
Few	16
Moderate	48
Many	25
Dense	11
Ellipsoid zone, *N*	
Integrity	38
Dot-like irregularity	41
Partial irregularity	18
Lack of continuity	3

*N* = number of eyes.

**Table 2:** Correlation analysis of OCT parameters and BCVA in different subgroups.

OCT parameters	Total		DME with SRF		DME without SRF	
	*N* = 100		*N* = 38		*N* = 62	
	Correlation coefficient (*r*)	*P* value	Correlation coefficient (*r*)	*P* value	Correlation coefficient (*r*)	*P* value
CMT	−0.550^+^	0.000^*∗∗*^	−0.472	0.003^*∗∗*^	−0.617^+^	0.000^*∗∗*^
Height of intraretinal cystoid	−0.526^+^	0.000^*∗∗*^	−0.088	0.617	−0.698^+^	0.000^*∗∗*^
Density of HRF	−0.277^−^	0.005^*∗∗*^	−0.271	0.115	−0.274^−^	0.031^*∗*^
Height of sponge-like retinal swelling	−0.411^+^	0.000^*∗∗*^	−0.240	0.159	−0.544^+^	0.000^*∗∗*^
EZ integrity	−0.501^+^	0.000^*∗∗*^	−0.216	0.130	−0.683^+^	0.000^*∗∗*^

^*∗*^ *P* < 0.05; ^*∗∗*^*P* < 0.01, ^+^moderate correlation, ^−^mild correlation.

**Table 3:** Results of multivariate linear regression analysis in different subgroups.

	Selected OCT parameters	Enrolled OCT parameters	Adjusted *R* square	*P* value	Coefficient	
					*β*	*P* value
Total	CMT	CMT	0.522	＜0.001	−0.482	0.000
	Height of intraretinal cystoid	Density of HRF			−0.184	0.059
	Height of sponge-like retinal swelling	EZ integrity			−0.447	0.000
	Density of HRF					
	EZ integrity					
DME	CMT	Height of intraretinal cystoid	0.605	＜0.001	−0.479	0.000
Without SRF	Height of intraretinal cystoid	Density of HRF			−0.167	0.100
	Height of sponge-like retinal swelling	EZ integrity			−0.388	0.004
	Density of HRF					
	EZ integrity					
DME with SRF	CMT	CMT	0.201	0.003	−0.472	0.003

*β*: standardized coefficients.

## Data Availability

Some or all data, models, or code generated or used during the study are available from the corresponding author upon request.

## Abbreviations

OCT: Optical coherence tomography
DME: Diabetic macular edema
CMT: Central macular thickness
HRF: Hyperreflective foci
EZ: Ellipsoidal zone
VA: Visual acuity
BCVA: Best-corrected visual acuity
DR: Diabetic retinopathy
PRP: Panretinal photocoagulation
DM: Diabetes mellitus
ETDRS: Early treatment diabetic retinopathy study
FFA: Fundus angiography
HRF: Hyperreflective foci
DRT: Diffuse retinal thickening
VEGF: Vascular endothelial growth factor
NPDR: Nonproliferative diabetic retinopathy.

## References

[1] Yau J. W., Rogers S. L., Kawasaki R. (2012). Global prevalence and major risk factors of diabetic retinopathy. *Diabetes Care*.

[2] Kempen J. H., O’Colmain B. J., Leske M. C. (2004). The prevalence of diabetic retinopathy among adults in the United States. *Archives of Ophthalmology*.

[3] Baskin D. E. (2010). Optical coherence tomography in diabetic macular edema. *Current Opinion in Ophthalmology*.

[4] Browning D. J., Glassman A. R., Aiello L. P. (2007). Relationship between optical coherence tomography-measured central retinal thickness and visual acuity in diabetic macular edema. *Ophthalmology*.

[5] Otani T., Kishi S., Maruyama Y. (1999). Patterns of diabetic macular edema with optical coherence tomography. *American Journal of Ophthalmology*.

[6] Sakamoto A., Nishijima K., Kita M., Oh H., Tsujikawa A., Yoshimura N. (2009). Association between foveal photoreceptor status and visual acuity after resolution of diabetic macular edema by pars plana vitrectomy. *Graefe’s Archive for Clinical and Experimental Ophthalmology*.

[7] Uji A., Murakami T., Nishijima K. (2012). Association between hyperreflective foci in the outer retina, status of photoreceptor layer, and visual acuity in diabetic macular edema. *American Journal of Ophthalmology*.

[8] Murakami T., Nishijima K., Sakamoto A., Ota M., Horii T., Yoshimura N. (2011). Association of pathomorphology, photoreceptor status, and retinal thickness with visual acuity in diabetic retinopathy. *American Journal of Ophthalmology*.

[9] Nalini M., Raghavulu B. V., Annapurna A. (2017). Correlation of various serum biomarkers with the severity of diabetic retinopathy. *Diabetes & Metabolic Syndrome: Clinical Research & Reviews*.

[10] Jain A., Saxena S., Khanna V. K. (2013). Status of serum VEGF and ICAM-1 and its association with external limiting membrane and inner segment-outer segment junction disruption in type 2 diabetes mellitus. *Molecular Vision*.

[11] Bringmann A., Reichenbach A., Wiedemann P. (2004). Patho mechanisms of cystoid macular edema. *Ophthalmic Research*.

[12] Scholl S., Augustin A., Loewenstein A., Rizzo S., Kuppermann B. D. (2011). General pathophysiology of macular edema. *European Journal of Ophthalmology*.

[13] Augustin A., Loewenstein A., Kuppermann B. D. (2010). Macular edema. general pathophysiology. *Macular Edema*.

[14] Zur D., Iglicki M., Busch C. (2018). OCT biomarkers as functional outcome predictors in diabetic macular edema treated with dexamethasone implant. *Ophthalmology*.

[15] Nadri G., Saxena S., Stefanickova J. (2019). Disorganization of retinal inner layers correlates with ellipsoid zone disruption and retinal nerve fiber layer thinning in diabetic retinopathy. *Journal of Diabetes and Its Complications*.

[16] Kang S. W., Park C. Y., Ham D.-I. (2004). The correlation between fluorescein angiographic and optical coherence tomographic features in clinically significant diabetic macular edema. *American Journal of Ophthalmology*.

[17] Bolz M., Schmidt-Erfurth U., Deak G., Mylonas G., Kriechbaum K., Scholda C. (2009). Optical coherence tomographic hyperreflective foci: a morphologic sign of lipid extravasation in diabetic macular edema. *Ophthalmology*.

[18] Alasil T., Keane P. A., Updike J. F. (2010). Relationship between optical coherence tomography retinal parameters and visual acuity in diabetic macular edema. *Ophthalmology*.

[19] Cho Y. J., Lee D. H., Kim M. (2018). Optical coherence tomography findings predictive of response to treatment in diabetic macular edema. *Journal of International Medical Research*.

[20] Bandyopadhyay S., Bandyopadhyay S. K., Saha M., Sinha A. (2018). Study of aqueous cytokines in patients with different patterns of diabetic macular edema based on optical coherence tomography. *International Ophthalmology*.

[21] Murakami T., Nishijima K., Akagi T. (2012). Optical coherence tomographic reflectivity of photoreceptors beneath cystoid spaces in diabetic macular edema. *Investigative Opthalmology & Visual Science*.

[22] De Benedetto U., Sacconi R., Pierro L., Lattanzio R., Bandello F. (2015). Optical coherence tomographic hyperreflective foci in early stages of diabetic retinopathy. *Retina*.

[23] Domalpally A., Ip M. S., Ehrlich J. S. (2015). Effects of intravitreal ranibizumab on retinal hard exudate in diabetic macular edema: findings from the RIDE and RISE phase III clinical trials. *Ophthalmology*.

[24] Tang J., Kern T. S. (2011). Inflammation in diabetic retinopathy. *Progress in Retinal and Eye Research*.

[25] Coscas G., De Benedetto U., Coscas F. (2013). Hyperreflective dots: a new spectral-domain optical coherence tomography entity for follow-up and prognosis in exudative age-related macular degeneration. *Ophthalmologica*.

[26] Baumüller S., Issa P. C., Scholl H. P. N., Schmitz-Valckenberg S., Holz F. G. (2010). Outer retinal hyperreflective spots on spectral-domain optical coherence tomography in macular telangiectasia type 2. *Ophthalmology*.

[27] Schuman S. G., Koreishi A. F., Farsiu S., Jung S.-h., Izatt J. A., Toth C. A. (2009). Photoreceptor layer thinning over drusen in eyes with age-related macular degeneration imaged in vivo with spectral-domain optical coherence tomography. *Ophthalmology*.

[28] Muftuoglu I. K., Unsal E., Ozturker Z. K. (2017). Restoration of photoreceptors in eyes with diabetic macular edema. *European Journal of Ophthalmology*.

[29] Shen Y., Liu K., Xu X. (2016). Correlation between visual function and photoreceptor integrity in diabetic macular edema: spectral-domain optical coherence tomography. *Current Eye Research*.

[30] Yohannan J., Bittencourt M., Sepah Y. J. (2013). Association of retinal sensitivity to integrity of photoreceptor inner/outer segment junction in patients with diabetic macular edema. *Ophthalmology*.

[31] Eliwa T. F., Hussein M. A., Zaki M. A., Raslan O. A. (2018). Outer retinal layer thickness as good visual predictor in patients with diabetic macular edema. *Retina*.

[32] Chen Q., Tan F., Wu Y. (2018). Characteristics of retinal structural and microvascular alterations in early type 2 diabetic patients. *Investigative Ophthalmology & Visual Science*.

